# Effect of Cu Particle Cross-Contamination in AlSi10Mg Powder Feedstock: Tensile and Strain-Hardening Behaviour of Multi-Material Laser Powder Bed Fusion Parts

**DOI:** 10.3390/ma19163367

**Published:** 2026-08-07

**Authors:** Nikolaos Alexopoulos, Ioanna Giavrouta, Leonard Alberty, Max Horn, Ismail Ünsal, Georg Schlick

**Affiliations:** 1 Research Unit of Advanced Materials, Department of Financial Engineering and Management, University of the Aegean, 82100 Chios, Greece; 2 Fraunhofer Research Institute for Casting, Composite and Processing Technology IGCV, Am Technologiezentrum 10, 86159 Augsburg, Germany

**Keywords:** AlSi10Mg, CuCr1Zr, additive manufacturing, laser powder bed fusion, cross-contamination, quality index, strain hardening

## Abstract

**Highlights:**

- Cu contamination in AlSi10Mg powder feedstock mainly affects tensile elongation at fracture of the parts.
- Strain-hardening is essentially influenced by cross-contamination level, showing high strain-hardening exponents with increased contamination level.
- Overall tensile performance of the specimens is affected by the cross-contamination level; inclined printing at 45° is less influenced.

**Abstract:**

Cross-contamination during metal powder blending in multi-material laser powder bed fusion (PBF-LB/M) is a common production challenge and a key barrier to the wider industrial adoption of the process. In the present investigation, the effect of different CuCr1Zr foreign-particle cross-contamination rates of up to 5.0 wt.%, simulating different cross-contamination levels in an AlSi10Mg feedstock for PBF-LB/M, is examined. The resulting metallurgical features and tensile mechanical properties of the produced components were compared to those of reference specimens manufactured from uncontaminated powder. A microstructural analysis of CuCr1Zr contaminated samples revealed characteristic Cu-rich regions, demonstrating that the higher the level of cross-contamination is, the larger these regions are. Tensile yield stress is almost linearly increased with the contamination level while the opposite trend is noticed for tensile elongation at fracture. Two different stages of strain-hardening were noticed, with Stage I exhibiting a lower strain-hardening exponent, while higher strain-hardening exponents (>0.27) were noticed for Stage II, with the latter decreasing with increasing cross-contamination level. The tensile mechanical behaviour of PBF-LB/M specimens was evaluated for the first time with appropriate quality indices, which were initially developed for similar cast aluminium alloys. Overall, the quality index accounting for global tensile performance was decreased for all build directions with increasing cross-contamination level. Despite the lower quality index at the non-contamination level, the inclined (45°) printed specimens presented quality indices that were almost unaffected by the cross-contamination level.

## 1. Introduction

Additive manufacturing (AM) has emerged as an advanced and increasingly adopted manufacturing process that is able to produce near-net-shape components with reduced lead times and without the need for conventional moulds. It has attracted significant interest owing to its flexibility in part design, material efficiency, and suitability for complex geometries [1,2,3]. Recently, laser powder bed fusion (PBF-LB/M) has gathered substantial and growing interest across both industrial applications and academic research communities. This layer-wise powder melting process is considered highly promising, as it enables the production of components with excellent quality and performance, and it is applicable to a broad range of alloys. These benefits render AM especially appealing for high-performance applications in different fields, including the aerospace and automotive sectors.

AlSi10Mg is a cast aluminium alloy that is extremely popular in aerospace and automotive lightweight structures, because it offers a favourable strength-to-weight ratio together with appreciable corrosion resistance and excellent casting behaviour [4]. This alloy is additionally capable of age-hardening, as the Mg content facilitates the formation of *β*-type (Mg_2_Si) precipitates during both natural and artificial ageing treatments [5,6]. Considering the benefits of PBF-LB/M and AlSi10Mg’s attractive properties, this alloy has become a prominent candidate for additive manufacturing when evaluated in terms of its chemical composition, suitable PBF-LB/M process parameters, and response to post-processing treatments [7,8,9].

Multi-material laser powder bed fusion (MM-PBF-LB/M) is increasingly recognised as an advanced manufacturing technique, capable of delivering high-precision metallic parts while simultaneously reducing production time and enhancing resource efficiency and sustainability. The powder feedstock control (particle size distribution and morphology) as well as the PBF-LB/M process parameters strongly influence the mechanical properties of the final component [10]. However, cross-contamination between different metal powders is likely one of the key limitations hindering the broader industrial adoption of this emerging additive manufacturing technology. Even very low levels of cross-contamination, particularly in multi-material builds involving dissimilar metals, can significantly degrade mechanical performance, and modify the functional behaviour of the fabricated parts [11]. Producing bimetallic components through additive manufacturing is especially demanding in the automotive field, where many applications require depositing aluminium alloys onto copper or CuCr1Zr substrates, or the reverse material combination [12]. This study specifically examines how the presence of CuCr1Zr particles as contaminants affects the AlSi10Mg powder feedstock.

The PBF-LB/M processing parameters are also verified to exert a pronounced effect on microstructure and crystallographic texture, as well as the tensile properties of AlSi10Mg alloys. Relevant research [8,13,14] has shown that adjusting parameters like scanning strategy, hatch distance, layer thickness, and laser power can significantly modify the resulting anisotropy and enable the formation of tailored microstructures and textures by adjusting the thermal gradient and solidification behaviour. Post-processing is widely used to tailor the microstructure and improve the mechanical properties of PBF-LB/M-processed AlSi10Mg alloys. Wang et al. [15] showed that heat treatment has a great influence on the microstructural features: the equiaxed grain structure and Si particles became coarse, while the cellular structure was removed. Zhang et al. [6] concluded that post-heat treatment can decrease fatigue properties due to size and morphology changes in Si particles, while Zhou et al. [5] proposed that artificial ageing accelerates the formation of *β*″ precipitates, thereby markedly increasing hardness through precipitation-strengthening.

Earlier investigations have shown that the presence of powder contamination has a negative effect on the resulting microstructure of additively manufactured parts and leads to a deterioration of key tensile and fatigue mechanical properties. Wang et al. [16] employed several metal powder mixtures of Al and Cu, containing up to 40 wt.% Cu in the Al matrix, where it was noticed that the Al_2_Cu content within the Al matrix was essentially increased, resulting in an inhomogeneous metallic microstructure composed of Al-, Cu-, and Al_2_Cu-rich regions. Mei et al. [7] reported that Cu incorporation enhances the cooling rate of the melt and favours both the metastability of Si and the nucleation of eutectic Al-Si, ultimately producing a refined eutectic network. Together with solid solution-strengthening and precipitation-hardening, this notably increased the AlSi10Mg mechanical strength level. Horn et al. [17] examined how the CuCr1Zr particles present in AlSi10Mg feedstock influence the resulting microstructure and tensile mechanical properties, and it was revealed that cross-contamination leads to overall material embrittlement. The strain-hardening response is expected to be influenced by the presence of copper-rich contaminant particles by introducing localised microstructural heterogeneities, disrupting load-transfer mechanisms, and generating additional sites for stress concentration. In a recent review article, Wang et al. [18] concluded that cross-contamination is one of the largest technical challenges holding multi-material PBF-LB/M back and that it needs to be further investigated. Finally, in a recent dissertation by Horn [19], the cross-contamination in both mono-material and multi-material PBF-LB/M was examined, focusing on the quantification of cross-contamination, reconditioning of material blends and determination of permittable cross-contamination. It was shown that even for mono-material PBF-LB/M, the permittable cross-contamination needs to be investigated, as material changes lead to detrimental amounts of residual powder in PBF-LB/M machines, even if the cleaning protocols of manufacturers are employed.

In the present study, attention is directed towards evaluating how cross-contamination of the AlSi10Mg feedstock with CuCr1Zr foreign particles influences the material tensile response, with particular emphasis on the resulting strain-hardening behaviour, which represents the main novelty of the present article. Strain-hardening plays a central role in mechanical behaviour by governing the material’s capacity to plastically deform and has a direct influence on its strength, ductility, and damage tolerance. A first approach to cover the strain-hardening capacity of PBF-LB AlSi10Mg printed at different orientations was made by Crawford and Serjouei [20], who exploited the Hollomon and Voce methods for approximating the work-hardening behaviour of non-contaminated material. Building on that, the influence of CuCr1Zr powder cross-contamination on the strain-hardening behaviour of AlSi10Mg parts produced with PBF-LB/M is expected to be clarified to control plastic deformation mechanisms and overall tensile mechanical performance. Finally, the tensile properties obtained will serve as the basis for quantifying the overall “quality” of the additively manufactured specimens produced at the various cross-contamination levels under investigation. This will be performed for the first time and represents a novelty of the present investigation. Diagrams of the resulting quality level, together with the targeted mechanical properties, will be then analysed as a function of both build direction and contamination level.

## 2. Materials and Methods

**Figure 1:**
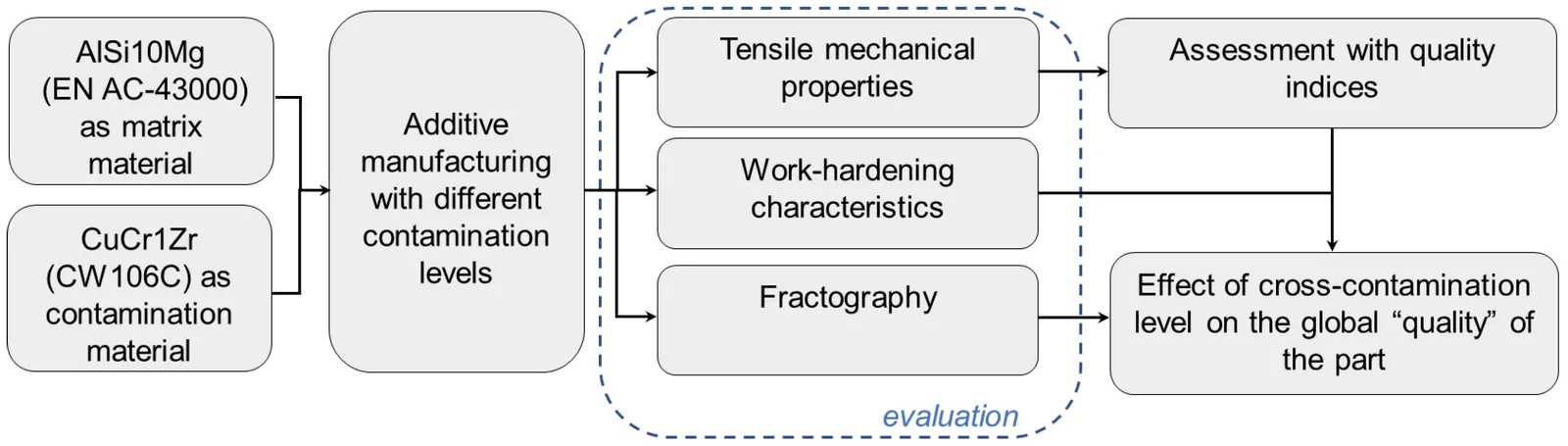
Flow diagram of the present investigation.

The current investigation focuses on the effect of the cross-contamination of automotive AlSi10Mg structures with CuCr1Zr particles that are being exploited for electrical/sensing purposes. The flow diagram of the investigation is illustrated in Figure 1, where the steps to achieve the input for the quantified effect of the cross-contamination are presented. Initially, different mixing scenarios were used, and the materials were additively manufactured in three (3) different building directions. After that, the tensile and work-hardening behaviour was evaluated to provide feedback on the global “quality” of the products, as expressed through the different indices proposed in the literature; the results will be analysed in the following. Finally, the results will allow for a systematic discussion of the building direction and the cross-contamination level of the investigated materials.

### 2.1. Materials

The product investigated in the present investigation is a high-performance automotive–power electronics sensing/cooling structure, and Al and Cu alloys were exploited. The Al-based alloy is primarily intended to act as the structural material, bearing and resisting the applied mechanical loads, while the Cu-based alloy can act as a functional material for electrical or thermal conduction.

The Cu alloy CW106C, commonly designated as CuCr1Zr, was chosen as the contaminant material. The powder chemical composition—according to produced specification—was as follows (wt.%): Cu: Balance; Cr: 0.69%; Fe: 0.005%; Si: <0.02%; Zr: 0.073%; and O: 0.003%. Introducing chromium (Cr) and zirconium (Zr) into the copper matrix enhances the mechanical strength, heat resistance, and wear resistance of the material, while only minimally compromising its inherent thermal and electrical conductivity. The CuCr1Zr powder, with a particle-size fraction of +20/−45 µm, was supplied by Schmelzmetall^©^ GmbH (Gurtnellen, Switzerland). The particle size distributions of both powder types were determined using a Mastersizer 3000 by Malvern Panalytical (Worcestershire, UK), and for the CuCr1Zr the powder Particle Size Distribution (PSD) was *d*_10_ = 20.0 µm, *d*_50_ = 32.2 µm, *d*_90_ = 45.0 µm.

Finally, an aluminium alloy standardised as EN AC-43000, commonly known as AlSi10Mg, was chosen as the matrix material. The powder chemical composition—according to the produced specification—was as follows (wt.%): Al: Balance; Si: 9.80%; Mg: 0.36%; Fe: 0.13%; and Sn: 0.01%. This alloy is a precipitation-hardenable casting material, owing to the formation of Mg_2_Si second-phase particles, and shows good electrical conductivity together with corrosion resistance, in accordance with DIN EN 1706. For the present investigation, metal powder from Nikon SLM Solutions AG (Lübeck, Germany) with +20/−63 μm classification and PSD *d*_10_ = 21.39 µm; *d*_50_ = 40.20 µm; and *d*_90_ = 69.76 µm was used.

**Figure 2:**
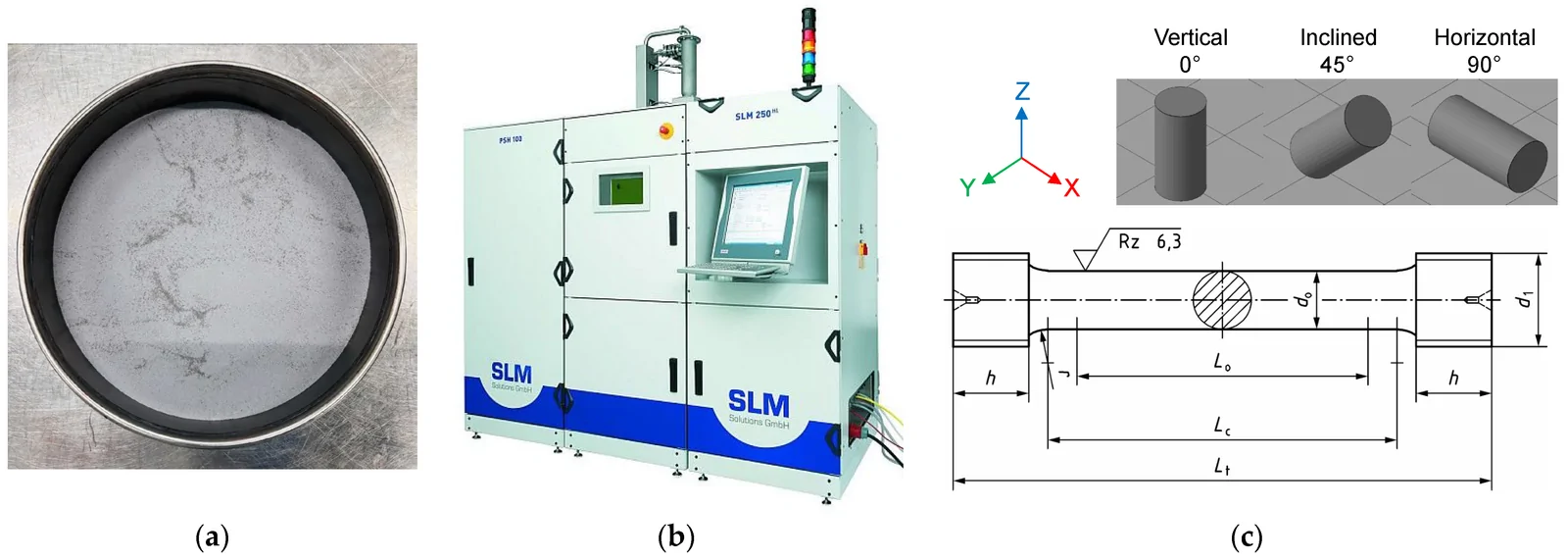
(**a**) Typical macro-photo of 3.0 wt.% cross-contamination of CuCr1Zr powder in AlSi10Mg feedstock, (**b**) SLM 250 HL PBF-LB/M machine used for printing the samples, and (**c**) investigated building directions and sketch of the tensile specimen.

To simulate realistic cross-contamination conditions, a distribution of foreign particles that is comparable to actual conditions in the process is targeted for the AlSi10Mg–CuCr1Zr mixture. To ensure comparability with the structural defects described in [17], the same contamination levels were chosen for this study. Because the qualitative behaviour at 0.5 wt.% and 1.0 wt.% contamination was nearly identical, the 1.0 wt.% condition was omitted from the present work; therefore, the respective specimens were not reproduced in this study, and it was decided to investigate the effect of higher possible cross-contamination levels. To this end, four (4) different cross-contamination levels of (a) 0.0 wt.%, as non-contaminated, (b) 0.5 wt.%, (c) 3.0 wt.%, and (d) 5.0 wt.% materials were finally analysed. For this, a closed mixing apparatus consisting of two hexagonal powder containers connected by a sealing clamp was designed. After ten minutes of manual rotation around all axes, powder mixing that resembles the actual process is achieved. Powder mixtures (Figure 2) were processed by Fraunhofer IGCV into the main tank of the SLM^©^ 250 HL PBF-LB/M machine from Nikon SLM Solutions (Lübeck, Germany) via a gravity-driven drop tube mounted on top of the machine.

### 2.2. Samples Building

Cubes with nominal dimensions of 10 mm × 10 mm × 10 mm were first fabricated and metallographically analysed. Semi-quantitative phase identification was performed using the Reference Intensity Ratio (RIR) method to evaluate the phase constitution of the printed material. Cylindrical specimens were produced in accordance with the DIN 50125–B4×20 standard, featuring a reduced section diameter of 4 mm. The tensile bars were initially additively manufactured as rods and subsequently machined to the final geometry according to the same standard, ensuring compliance with the prescribed dimensional tolerances. More specifically, the printed specimens had the following final geometry: total length *l*_total_ = 41 mm; reduced diameter *d* = 4.0 mm; and gauge length at the reduced cross-section *l*_gauge_ = 20 mm. A minimum of three (3) tensile specimens was produced for each build orientation examined (namely 0°, 45°, and 90° relative to the build direction) and for the different contamination levels investigated. The three (3) samples used in each case were selected to enable a statistically meaningful evaluation of the tensile mechanical response.

### 2.3. Material Characterisation

The tensile tests were conducted on a Zwick Roell (Ulm, Germany) UPM 50 kN loading frame according to DIN EN ISO 6892-1. During mechanical testing, axial load, axial crosshead deformation and strain measurements from the extensometer attached to the gauge length of the specimens were continuously recorded. The recorded data of the mechanical load and displacement of the gauge length values were then converted to engineering stress–engineering strain data, allowing for the assessment of the tensile and work-hardening behaviour. The following mechanical properties were evaluated as follows: conventional yield stress *R*_p0.2%_ was obtained using the 0.2% offset method, ultimate tensile strength *R*_m_ was defined as the maximum stress recorded, uniform elongation *A*_g_ limit was recorded as the start of necking, coinciding with the strain value at *R*_m_ point, tensile elongation at fracture *A*_f_ was calculated as the fracture strain with excluded elastic deformation, and finally strain energy *W* accounted for the energy per volume (integration of the σ-ε curve) until fracture. The tensile nominal axial stress–nominal axial strain curves, from the yield point up to the onset of uniform elongation, were subsequently analysed to characterise the material’s work-hardening behaviour.

The fracture surfaces were analysed by scanning electron microscopy (SEM) using a Hitachi TM3030Plus (Tokio, Japan) instrument. Backscattered electron (BSE) imaging was employed to obtain compositional contrast, while secondary electron (SE) imaging was used to capture detailed topographical features of the fracture surfaces.

### 2.4. Background in Quality Assessment

Achieving an optimal balance between mechanical strength and tensile ductility remains a critical issue when developing new aluminium alloys, especially in castings, and can be extended to produce additively manufactured alloys and products. In this regard, the mechanical test results were exploited as input parameters to qualitatively define the “quality” of castings/materials by exploiting the “quality index” concept, which will be analysed in the following. This concept, originally introduced by Drouzy et al. [21], evaluates the influence of chemical composition and thermal treatment variations in cast AlSiMg aluminium alloys on the casting “quality”, calculated based on their tensile mechanical properties. Empirical Equation (1) was introduced to minimise experimental effort while comprehensively evaluating the tensile strength and ductility potential of materials:

*Q* = *R*_m_ +150·log(*A*_f_), (1)

where *R*_m_ denotes the ultimate tensile strength and *A*_f_ represents the tensile elongation at fracture. Drouzy’s index incorporates two distinct terms: the former provides insight into the mechanical strength level, while the latter quantifies the elongation ability (expressed via the logarithm of elongation at fracture), thereby enabling a single numerical value to encapsulate the overall tensile mechanical performance capability. The empirical constant of 150 was determined through extensive experimentation, testing and evaluation, and is assumed to be a fixed material constant for A357 (AlSiMg) cast aluminium alloys.

However, when these alloys are intended for modern lightweight structures, they must exhibit high damage tolerance capability. Consequently, their quality assessment should incorporate quantitative indices that also correspond to damage tolerance. In this framework, Alexopoulos and Pantelakis [22] introduced a quality index that incorporates damage-tolerance mechanical behaviour into a single quantitative parameter, *Q*_0_, which is further scaled by a dimensionless factor, *K*_D_, to account for the inherent scatter in the material’s mechanical properties. Thus, quality index *Q*_D_ accounting for damage tolerance capabilities is formulated as follows:

*Q*_D_ = *K*_D_ · *Q*_0_, (2)

where *K*_D_ represents the statistical scatter exhibited by the material’s mechanical properties, and *Q*_0_ characterises the material’s tensile mechanical performance. Analogous to the quality index of Drouzy et al. [21], *Q*_0_ encompasses both mechanical strength and tensile ductility components, summing them to yield baseline quality, expressed as follows:

*Q*_0_ = *R*_p_ + 10 · *W*, (3)

where *R*_p_ refers to the tensile yield stress and *W* to the tensile strain energy density, defined as the area under the engineering stress–engineering strain curve. The latter mechanical property (*W*) was selected to be accounted for in the quality index calculation as it was determined to more effectively capture the material’s damage-tolerance characteristics. More specifically, Sih and Jeong [23] established a direct correlation between energy density and plane–strain fracture toughness *K*_Ic_. The empirical constant of 10 corresponds to a characteristic value of the *R*_p_/*W* ratio of already-optimised structural aluminium alloys, as established by Alexopoulos [24]. Finally, the *Κ*_D_ factor was determined as follows:

(4) $$K_{D}=\left(\frac{R_{pi}}{R_{pmax}}+\;\frac{W_{i}}{W_{max}}\right)\;\leq 2,$$

where *R*_pi_ denotes the yield stress of the *i*-th specimen, *R*_pmax_ is the maximum yield stress obtained within the same test series, *W*_i_ is the energy density corresponding to the *i*-th specimen, and *W*_max_ represents the maximum energy density measured in that series.

## 3. Results and Discussion

This section presents the metallographic and mechanical test results and provides valuable discussion regarding the cross-contamination effect of the samples.

### 3.1. Macroscopic Characterisation

**Figure 3:**
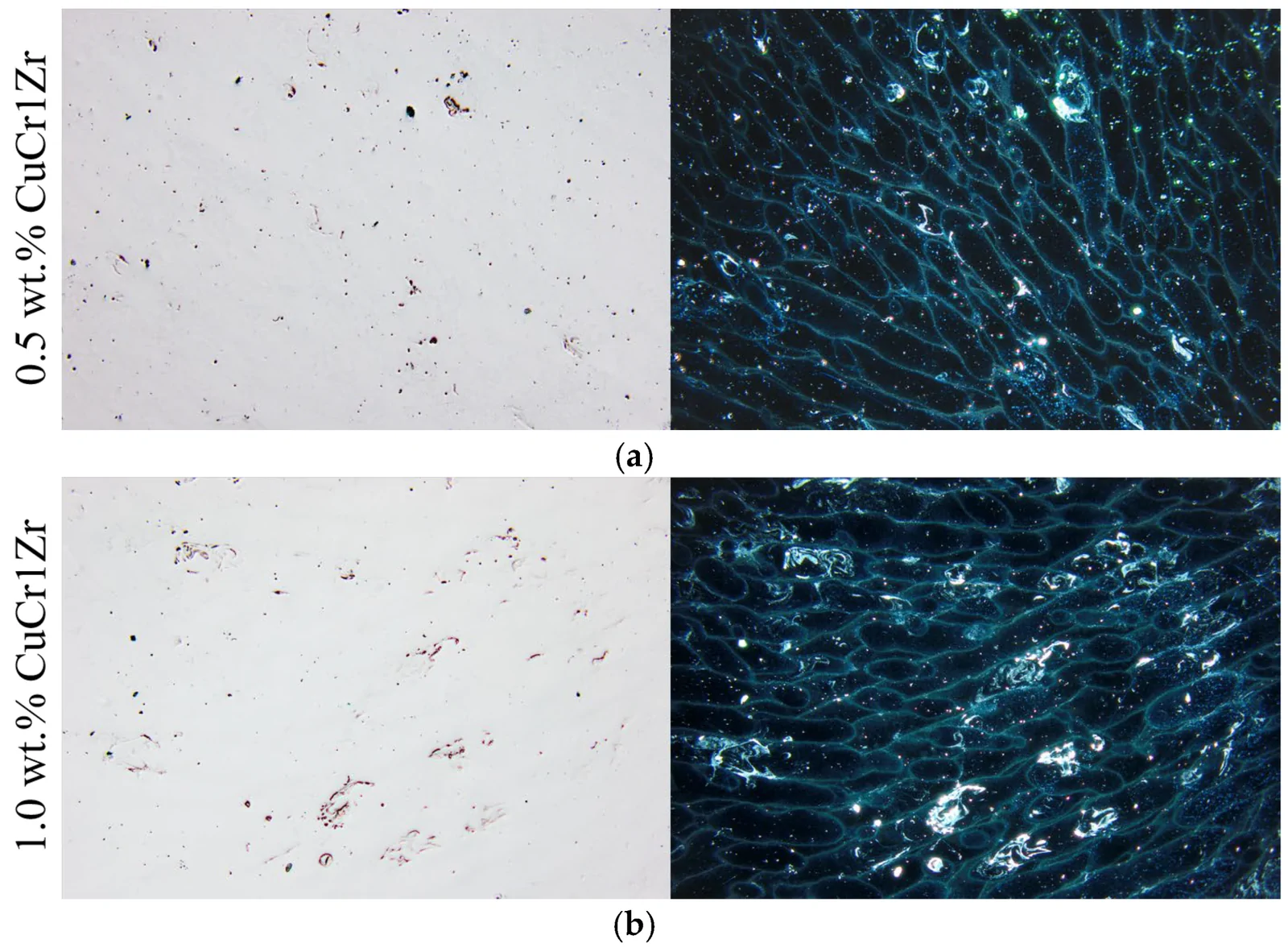

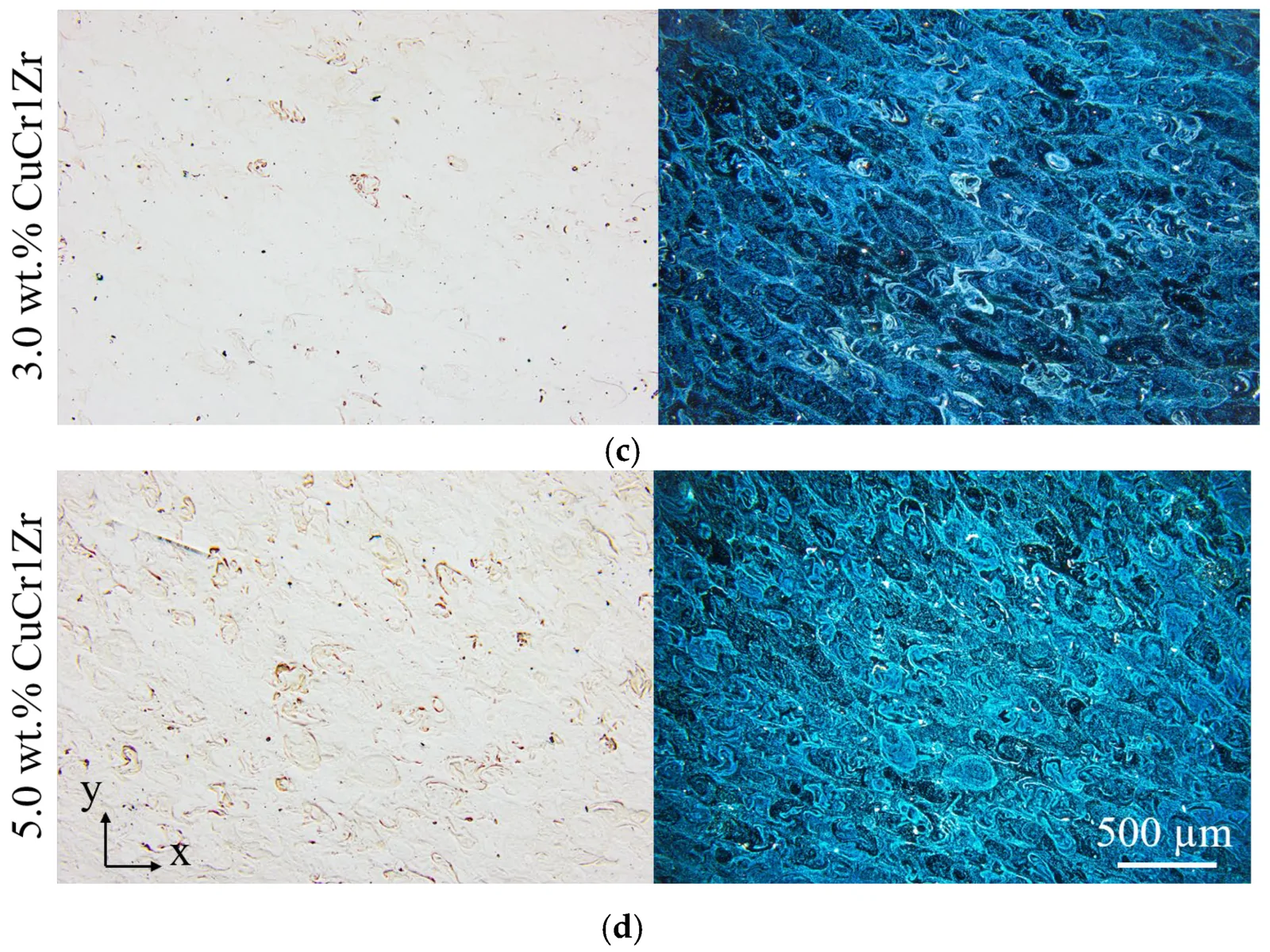
Light optical microscopy images (bright/dark-field) for the different CuCr1Zr contamination levels, namely (**a**) 0.5 wt.%, (**b**) 1.0 wt.%, (**c**) 3.0 wt.% and (**d**) 5.0 wt.%, in AlSi10Mg powder [25]. All images have the same scale.

A qualification of cross-contaminated materials of several contamination levels, mixed via the same process as in this study, is performed in [25]. The manufacturing process, including the used powder, the PBF-LB/M-machine and process parameters, is identical to the present work. For the analysis, light microscopy was performed on cross-sectional surfaces using bright-field and dark-field microscopy. Figure 3 displays the obtained light optical microscopy images of the investigated specimens with different cross-contamination levels. The images show that the powder was uniformly blended, yielding a level of mixing comparable to the cross-contamination expected in an actual multi-material PBF-LB/M process.

### 3.2. Mechanical Performance

**Figure 4:**
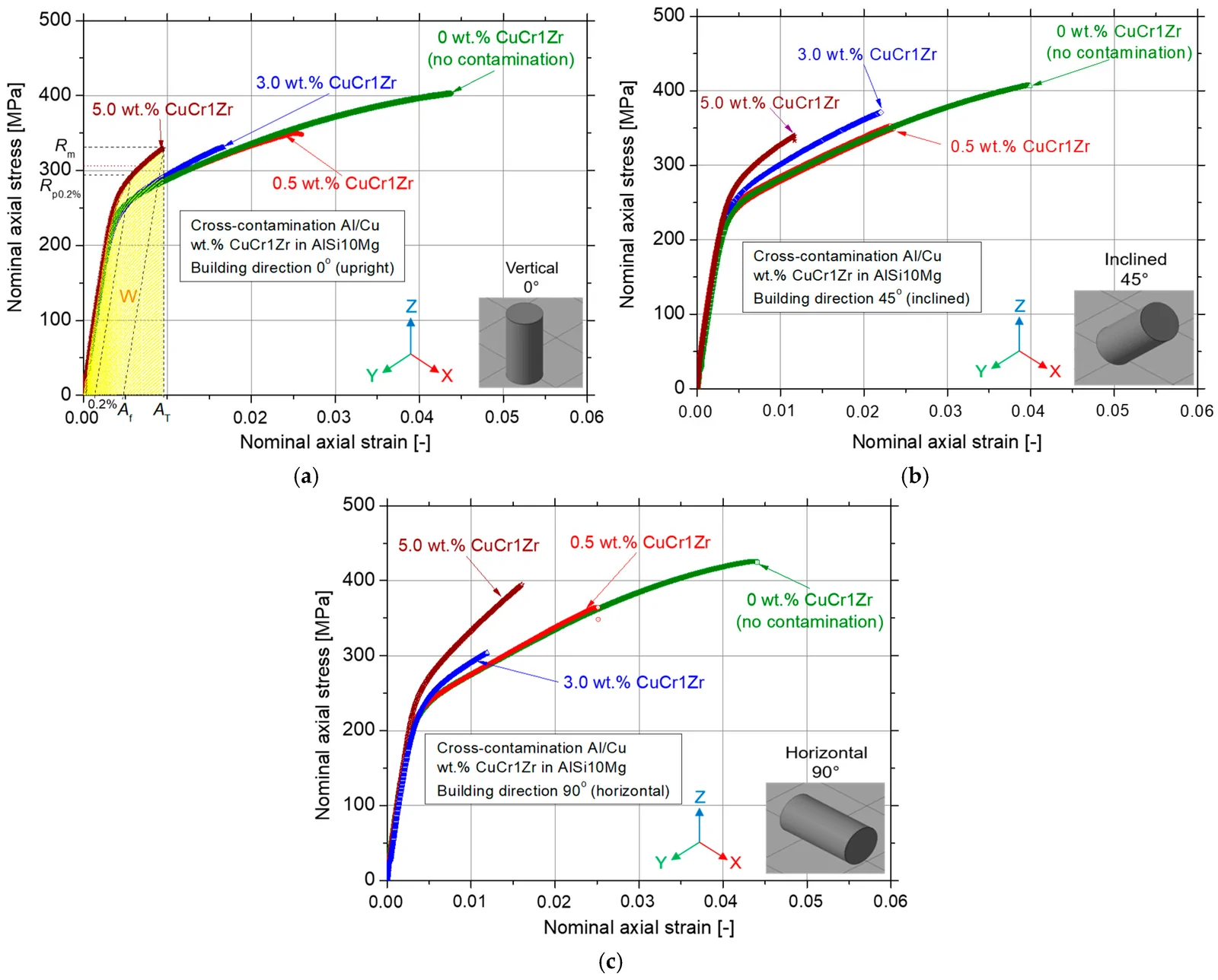
Representative tensile nominal axial stress–nominal axial strain curves of the additively manufactured AlSi10Mg specimens built in (**a**) upright (0°), (**b**) inclined (45°), and (**c**) horizontal (90°) directions for the investigated CuCr1Zr cross-contamination levels.

Figure 4a presents representative tensile nominal axial stress–nominal axial strain flow curves for additively manufactured AlSi10Mg specimens built in a vertical (0°) orientation, obtained at various CuCr1Zr powder cross-contamination levels; the other two (2) build directions can be seen in Figure 4b and Figure 4c, respectively. Specimens with high cross-contamination levels (e.g., 3.0 wt.% and 5.0 wt.%) exhibit a distinctly steeper tensile stress–strain behaviour and fail at lower strains, reflecting premature fracture. This behaviour points to the progressively increased embrittlement of the material as the degree of powder cross-contamination increases. Furthermore, the calculation of strain energy *W* from the stress–strain diagram is graphically presented in the first diagram.

**Figure 5:**
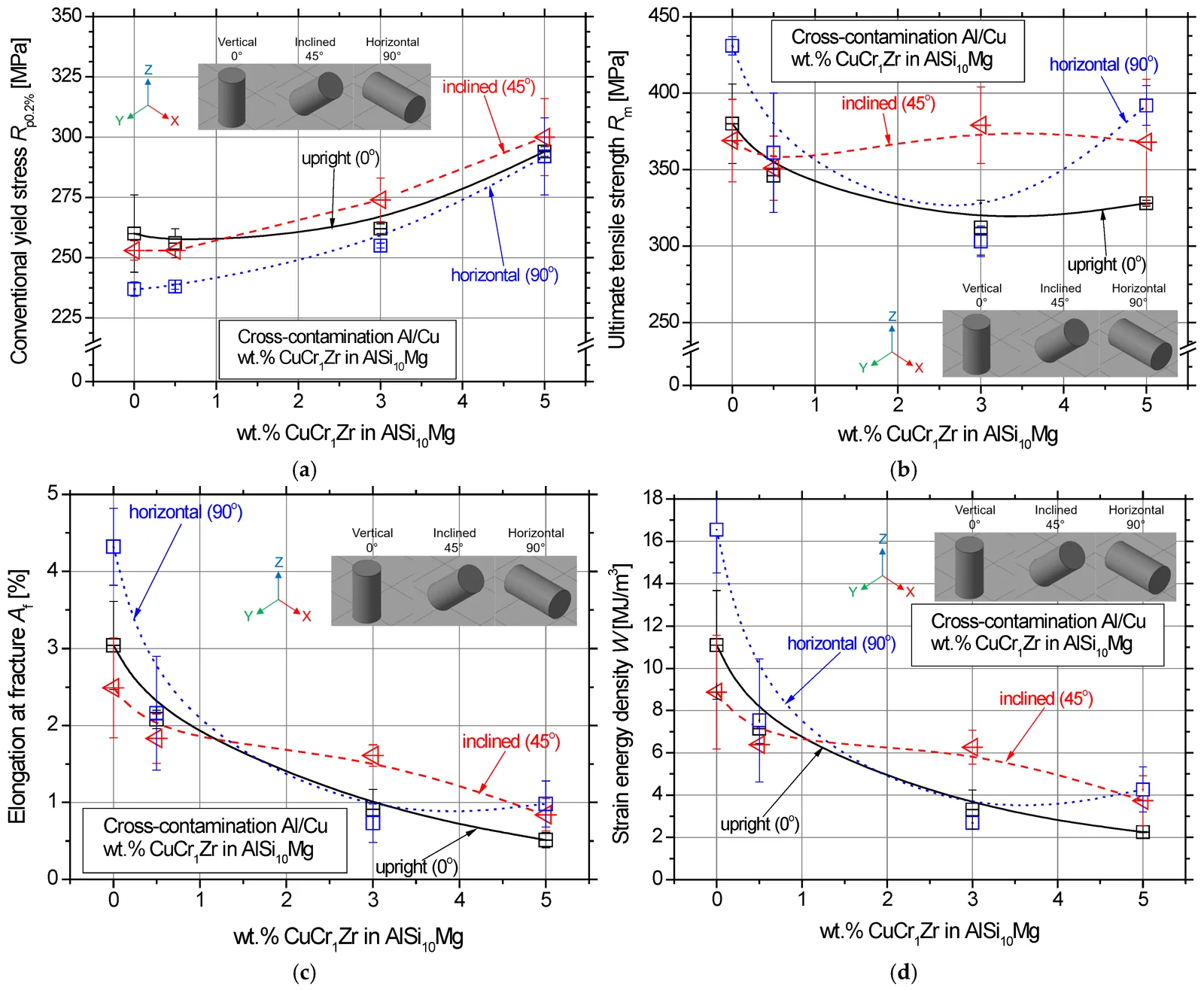
Effect of CuCr1Zr contamination on the tensile mechanical properties of additively manufactured AlSi10Mg specimens printed in different orientations (upright (0°), inclined (45°), and horizontal (90°): (**a**) conventional yield stress *R*_p0.2%_, (**b**) ultimate tensile strength *R*_m_, (**c**) elongation at fracture *A*_f_ and (**d**) strain energy density *W*.

The effect of cross-contamination on the key quasi-static tensile properties—*R*_p0.2%_, *R*_m_, *A*_f_, and *W*—is summarised in Figure 5a to Figure 5d, respectively. All graphs present the respective mechanical properties derived from the tensile tests, where a minimum of three (3) specimens were tested for each condition to obtain statistically reliable results in terms of average value and standard deviation. The tensile mechanical properties were evaluated from the tensile flow curves and for the three (3) different build orientations (0°, 45°, and 90°). The results of conventional yield stress are illustrated in Figure 5a, showing a gradual increase with contamination level. At low contamination levels (0 to 0.5 wt.%), conventional yield stress ranges roughly between 235 and 255 MPa, whereas at higher contamination levels it increases to approximately 290 to 305 MPa. Specimens produced in horizontal (90°) and inclined (45°) directions exhibit an almost linear *R*_p0.2%_ increase with increasing contamination level, while specimens produced in the upright direction increase at a lower rate. Additionally, the highest yield stress values at 0 to 0.5 wt.% contamination levels appear in the upright (0°) direction, whereas at higher contamination levels (3.0 to 5.0 wt.%), the highest yield stress values are obtained in the inclined (45°) direction. Similar results were noticed by Zhao et al. [26], where tensile tests demonstrated that build orientation (0°, 45°, and 90°) significantly influenced the yield stress and strain-hardening of an AlSi10Mg alloy fabricated by PBF-LB/M.

From a metallurgical perspective, the binary aluminium–copper system can form several distinct precipitations, e.g., *θ*-type (Al_2_Cu) or *S*-type (Al_2_CuMg) particles, where copper serves as the primary alloying element in aluminium alloys, facilitating precipitation-hardening. The Al matrix can dissolve up to 5.56 wt.% Cu to form a solid solution; once this solubility limit is exceeded, the *θ*-Al_2_Cu intermetallic phase begins to form, and at 53.5 wt.% Cu the alloy exists entirely as the *θ* phase. In this context, CuCrZr cross-contamination can increase yield stress either by promoting the formation of precipitates, such as the *θ* phase, or through solid-solution-strengthening arising from the incorporation of relatively large Cu particles into the aluminium matrix.

Copper contamination appears to exert differential effects depending on the specific tensile mechanical property under consideration, such as ultimate tensile strength *R*_m_. Figure 5b compiles *R*_m_ values for all investigated cross-contamination levels, showing that in the inclined (45°) build orientation the *R*_m_ remains nearly constant, exhibiting only minor fluctuations. In the other two (2) building directions, it appears that two trends emerge. From the lower range of 0 to 3.0 wt.% there is a significant linear *R*_m_ decrease with increasing contamination level, especially in the upright (0°) direction, where *R*_m_ drops from 420 MPa to approximately 300 MPa (a decrease of more than 100 MPa). At the higher examined 5.0 wt.% contamination level, *R*_m_ recovery is observed (which is greater in the horizontal (90°) direction), likely due to the intermetallic phases formed by the high copper content.

In contrast, increasing levels of cross-contamination led to a reduction in the elongation at fracture *A*_f_ across all build orientations examined, as graphically presented in Figure 5c. The inclined (45°) direction appears to follow a linear *A*_f_ decrease in relation to the increasing contamination level, whereas the other two building directions show a continuous decrease (closer to exponential decrease). It can be easily noticed that the horizontal (90°) building direction exhibits the highest tensile elongation at fracture values in almost all investigated contamination levels.

Finally, the strain energy density *W* results are graphically illustrated in Figure 5d, where the largest decrease corresponds to the horizontal building direction, which starts at approximately 16 MJ/m^3^ and drops to about 4 MJ/m^3^ (~75% decrease), while the smallest decrease is observed for the inclined building direction, which starts at 8 MJ/m^3^ and ends up at 4 MJ/m^3^ (~50% decrease). The upright building direction, in turn, shows a decrease starting from 12 MJ/m^3^ and ending at 2 MJ/m^3^ (about 83% decrease).

In late 1950s and early 1960s, pioneering studies were performed [27,28,29] on copper and other face-centred cubic (FCC) single crystals regarding strain-hardening. All of them noted three different stages of strain-hardening and subsequent review articles linked these stages to dislocation mechanisms (e.g., Nabarro et al. [30]; Kocks and Mecking [31]), with the latter providing a comprehensive historical overview of the work-hardening theory. Stage I, commonly referred to as “easy glide,” is greatly influenced by crystal orientation and manifests exclusively during single slip in mono-crystals. It is absent when multiple slip systems are activated from the outset. Stage II, designated “athermal work-hardening”, exhibits linear hardening characterised by an increased work-hardening rate. This stage is strongly orientation-dependent and largely insensitive to external variables (athermal), and serves as an asymptote for low-strain multiple-slip deformation. Stage III, also known as “dynamic recovery,” features a monotonic decrease in the strain-hardening rate and exhibits strong sensitivity to both temperature and strain rate. Theoretical considerations suggest that the strain-hardening rate attains a plateau, at which point dynamic recovery counterbalances dislocation accumulation. Stage III is strongly material-dependent and has been correlated [31,32,33] with the normalised stacking fault energy. It is widely recognised that the onset of Stage III constrains the progression of Stage II, especially in high-stacking-fault energy materials, as well as at elevated temperatures.

**Figure 6:**
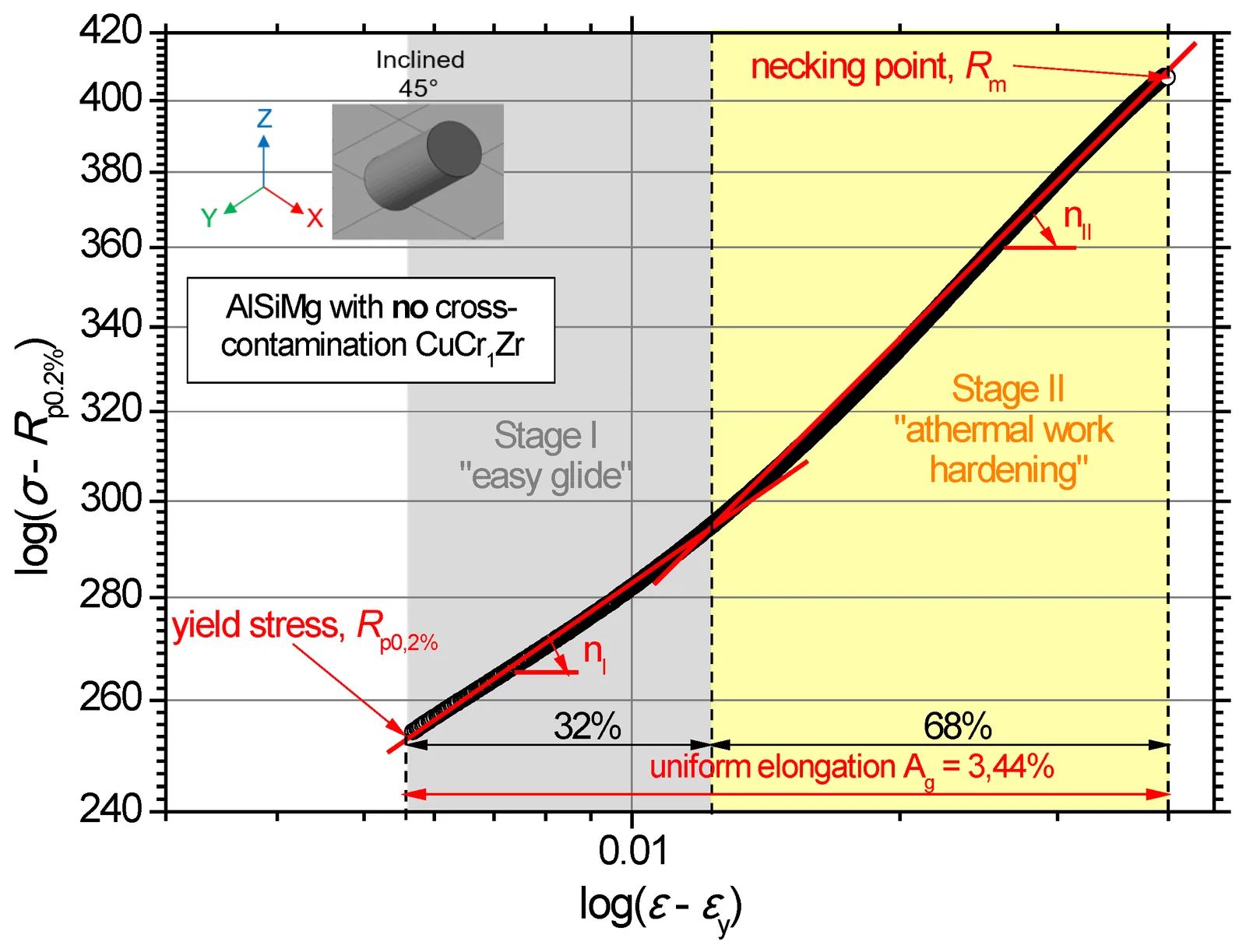
Graphical representation of the strain-hardening behaviour of an AlSi10Mg specimen built in an inclined (45°) direction without CuCr1Zr cross-contamination.

The experimental curve presented in Figure 6, plotted on a log–log scale, depicts plastic strain exclusively for a reference, non-contaminated additively manufactured AlSi10Mg tensile specimen (built in an inclined (45°) direction), with the initial elastic region (up to 0.2% elastic strain) omitted from the figure. Linear regression fitting (red lines) was used for the two identified strain-hardening stages, while the intersection of the two fitted lines provides an approximation of the end of the first strain-hardening stage and the beginning of the other. The strain-hardening rate, represented as the first derivative of the experimental tensile nominal stress–nominal strain flow curve, is clearly discernible and annotated to delineate the distinct stages of work-hardening. In this regard, strain-hardening exponents (*n*_I_ and *n*_II_) were calculated as the slope of the fitted lines from the conventional yield stress point (*R*_p0.2%_) to the necking point (*R*_m_) for the present example, which consisted of an AlSi10Mg specimen built in an inclined (45°) direction without CuCr1Zr cross-contamination.

In the present investigation, the presence of two (2) distinct work-hardening stages (with the respective strain-hardening exponents *n*_I_ and *n*_II_) was noticed. Stage III was not confirmed in the present investigation, possibly due to pre-mature fracture due to dispersoids, particles and voids. In this regard, the research presented in the bibliography [34,35,36] on additively manufactured aluminium alloys confirmed the absence of the third stage of strain-hardening. As a general observation, at both low and intermediate CuCr1Zr cross-contamination levels, the first two stages of strain-hardening can be clearly noticed in the tensile flow curve, spanning the region between the conventional yield point and the onset of macroscopic necking. At elevated cross-contamination levels, brittle behaviour can be noticed, with fracture initiating prematurely within the first stage of work-hardening.

**Figure 7:**
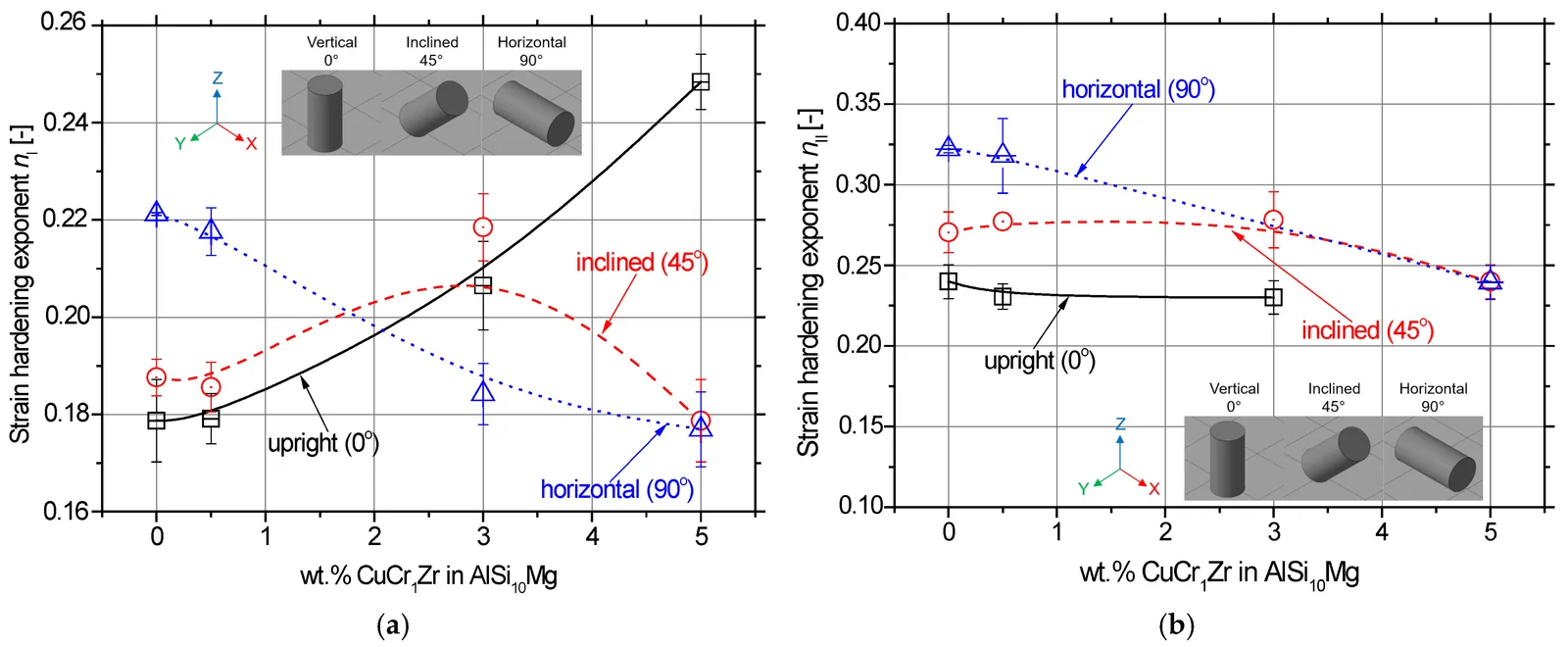
Evaluation results of tensile strain-hardening exponents (**a**) *n*_I_ (**b**) *n*_II_ from the first two strain-hardening stages.

Figure 7a and Figure 7b present the variation in *n*_I_ and *n*_II_ strain-hardening exponents, respectively, as a function of the different CuCr1Zr contamination levels. Figure 7a shows that *n*_I_ has a linear increase that ranges from 0.18 to 0.25 in the upright direction, while in the inclined direction, *n*_I_ increases up to 3.0 wt.% contamination levels, but then decreases sharply, falling below the baseline at the 5.0 wt.% contamination level. In contrast, in the horizontal direction, *n*_I_ appeared to decrease continuously in a linear manner, from 0.22 down to 0.18 at the highest contamination level. It must be noticed that Stage I (easy glide), is majorly influenced by crystal orientation and this linear trend can possibly be attributed to the build-up direction, as the deposition of the material thermally influences the lower deposited material.

As anticipated, linear Stage II exhibits a higher strain-hardening rate compared to the preceding stage. A similar decreasing trend of *n*_II_ in Figure 7b is observed in all directions, with the largest reduction in the horizontal direction, the smallest in the upright direction and an intermediate decrease in the inclined direction, which remains nearly constant and decreases only at the highest contamination level.

**Figure 8:**
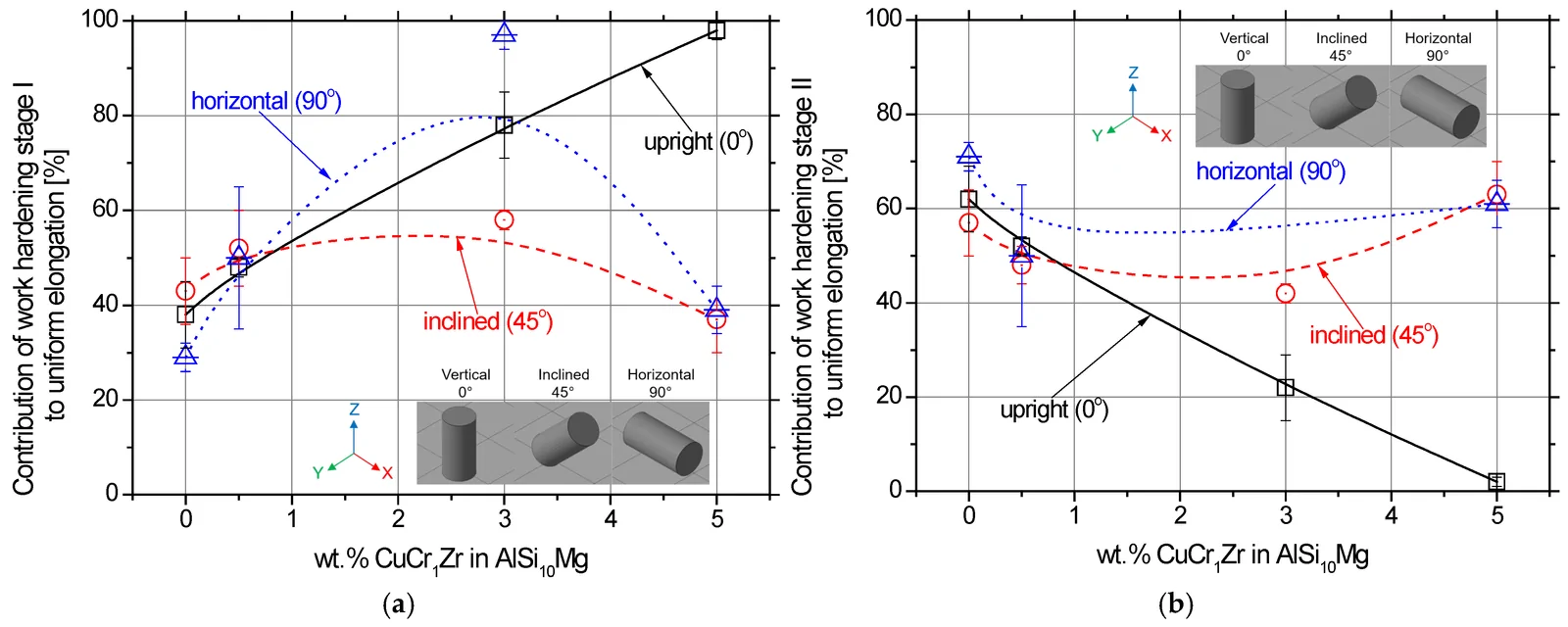
Contribution (or extent) of the two (2) work-hardening stages (**a**), *S*_I_ of Stage I and (**b**) *S*_II_ of Stage II, to uniform elongation at different CuCr1Zr cross-contamination levels in AlSi10Mg tensile specimens built in different orientations (upright (0°), inclined (45°), and horizontal (90°)).

The extent (or duration) of plastic elongation associated with each strain-hardening stage was expressed as a normalised value with respect to the specimen’s total uniform elongation. Figure 8a shows the contribution of strain-hardening stage I to uniform elongation. For the upright direction (0°), the contribution of Stage I increases significantly with increasing contamination level. It rises from approximately 40% at 0 wt.% contamination level to nearly 100% at a 5.0 wt.% contamination level, indicating that the early stage of work-hardening becomes increasingly dominant in controlling the uniform deformation. It should be noted that the diagram employs a logarithmic scale; consequently, Stage I persists for nearly 40% of the total and the remaining 60% is Stage II of the total uniform elongation, despite the latter being visually abbreviated in the figure.

In the horizontal direction (90°), Stage I extends from 30% to nearly 100% of the total uniform elongation at the 3.0 wt.% contamination level but then drops sharply to around 40% at the 5.0 wt.% contamination level. This suggests that at higher contamination levels the importance of Stage I decreases. For the inclined direction (45°), the contribution remains relatively stable, at around 50 to 60%, up to 3.0 wt.% contamination levels, followed by a decrease to approximately 40% at the 5.0 wt.% contamination level. Overall, the results indicate that Stage I becomes increasingly dominant in the upright direction, while in the other two (2) directions its contribution is more moderate and decreases at higher percentages of cross-contamination. The experimental data indicate that, in all cases, Stage I exhibits a low work-hardening rate, which the literature attributes to the minimal accumulation of dislocation line length. The literature reports [37,38] that during Stage I, the dominant dislocation storage mechanism is the formation of dislocation dipoles, in which parallel edge dislocations of opposite sign arrange into a stable configuration. Argon and East [39] proposed a statistical description of Stage I strain-hardening, in which dislocations are progressively “captured” into dipolar or multipolar arrangements.

Stage II exhibits substantially higher strain-hardening rates compared to the preceding stage. Several principal mechanisms—secondary slip, forest dislocations, and areal glide—are also proposed to contribute during Stage II. The areal glide model appears to offer a more satisfactory description of Stage II than the existing models reported in the literature [40,41,42]. A mobile dislocation can induce macroscopic yielding, despite leaving small concave loops in its wake as it navigates around local “hard spots”—in this case, second-phase precipitates or dispersoids. Precipitates distributed within the matrix induce fluctuations in dislocation density; consequently, their size and distribution—which vary across different cross-contamination levels—is extremely essential in determining the travel distance of mobile dislocations and their ability to bypass these obstacles. The second-phase particles are either penetrable (e.g., Guinier–Preston–Bagaryatsky (GPB) zones and *S*″-type precipitates) or impenetrable (e.g., *S*′- and *S*-type precipitates) by dislocation, depending on their size, as extensively documented [43,44]. For penetrable precipitates, dislocation accumulation results in particle shearing, thereby enabling continued dislocation motion along the slip plane. For impenetrable precipitates, dislocation tangles arise from the build-up of concave glide loops around these obstacles on the slip plane, and they relax once a critical pile-up configuration is attained. Consequently, the concave loops formed on the primary slip system are converted into dislocation density on secondary slip systems, which in turn promotes areal glide on these alternative slip systems. The critical factor in areal glide appears to be the precipitates spacing, which is inverse proportional to *n*_II_.

The experimental results presented in Figure 8b indicate that the extent of Stage II to uniform elongation varies with building direction and the cross-contamination level. Stage II duration is markedly reduced to 0.5 wt.% cross-contamination, coinciding with the formation of semi-coherent *β*′′ precipitates for all building directions. For the upright direction (0°), the contribution of Stage II decreases significantly, dropping from about 60% to approximately 20% at the 3.0 wt.% cross-contamination level. In contrast, for the horizontal direction (90°) the contribution remains relatively high (around 50 to 70%), indicating that Stage II plays a more significant role in the deformation behaviour for this printing direction. This can be explained as the higher cross-contamination level allows for *S*-type particles to be coarser and higher in number. In this regard, particle shearing becomes unfeasible, meaning that particles loop the dominant dislocation interaction mechanism. This results in a substantial increase in Stage II strain duration to approximately 60% of the total uniform elongation in the horizontal building direction. This effect was also noticed for the inclined orientation (45°), where an increase in Stage II from roughly 40% to 60% is evident.

Overall, the results highlight a strong dependence of the work-hardening behaviour on the printing orientation of the specimens. With increasing CuCr1Zr contamination, Stage I becomes more dominant in the upright direction, while Stage II progressively decreases in importance in the same direction.

### 3.3. Fractography

**Figure 9:**
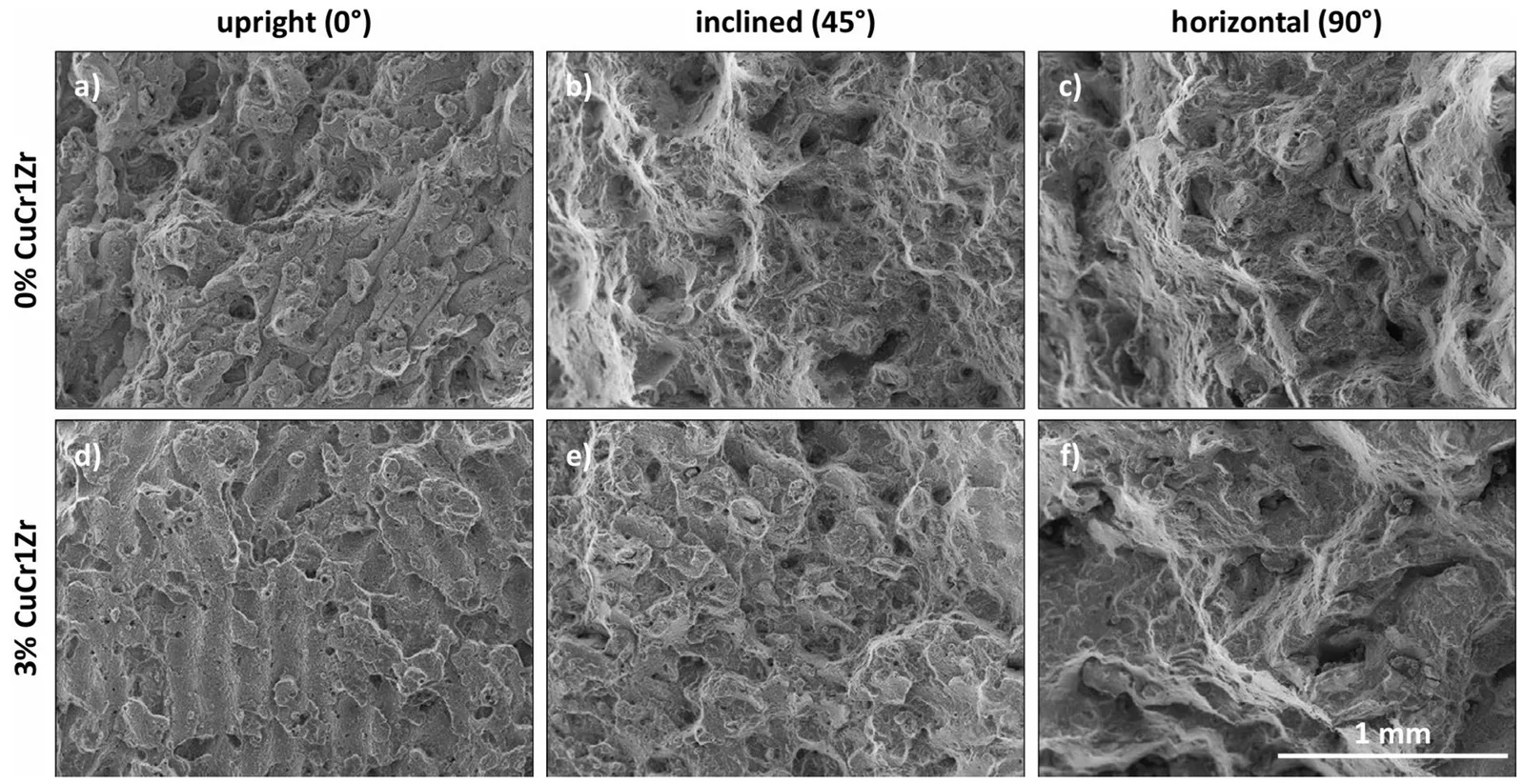
Fractographic SEM images of AlSi10Mg specimens built with different orientations (upright (0°), inclined (45°), horizontal (90°), for 0.0 wt.% CuCr1Zr (**a**–**c**) and 3.0 wt.% CuCr1Zr (**d**–**f**) cross-contamination [17].

All observed fracture surfaces exhibited predominantly brittle characteristics, with no discernible evidence of necking. Inclined samples (45°) exhibit a propensity for shear fractures oriented at 45°, whereas upright samples (0°) predominantly display cleavage fractures along horizontal planes. Horizontally built samples exhibit fractures in both orientations, with a slight predominance of angled fracture surfaces. The systematic nature of observed fracture types indicates that they originate from intrinsic material microstructure rather than the shear stresses induced by clamping during tensile mechanical testing. Macroscopic fracture surface morphology and general fracture types remain unaffected by contamination levels, as exemplified in Figure 9 for CuCr1Zr alloys at 0 wt.% and 3.0 wt.% contamination. Upright samples across all contamination levels were observed to contain melt track remnants, potentially indicative of bonding defects that account for their propensity toward cleavage fractures. The overall microstructure consistently exhibits transgranular cleavage or even shear-type fracture with low tensile ductility, regardless of build orientation or cross-contamination level.

**Figure 10:**
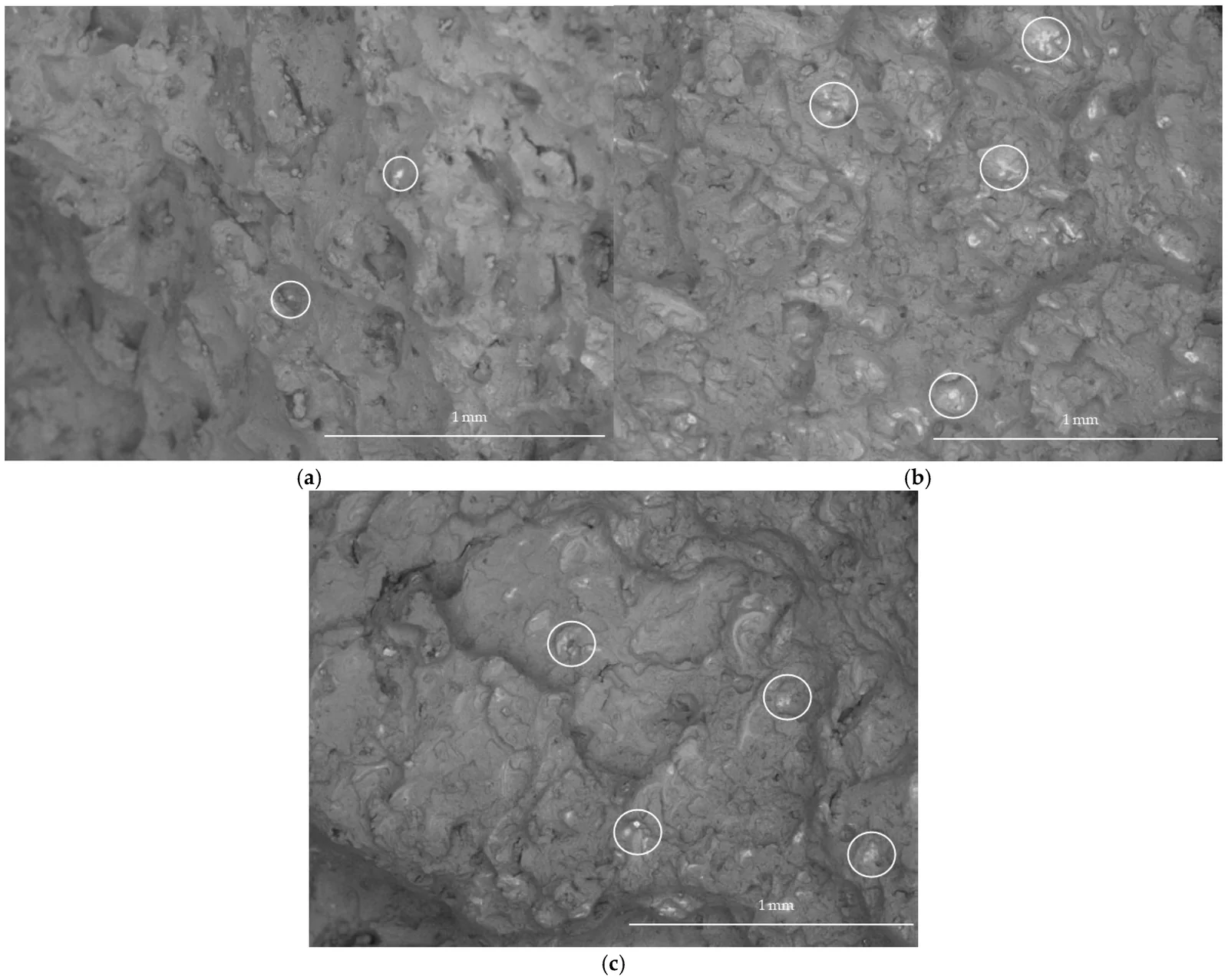
Fractographic SEM images of AlSi10Mg specimens (back-scattered electrons) with (**a**) 0.5 wt.%, (**b**) 3.0 wt.% and (**c**) 5.0 wt.% CuCr1Zr contamination built in an inclined (45°) orientation [17]. Copper-rich precipitates/particles exhibit enhanced brightness, attributable to their elevated material density (denoted by circles).

Figure 10 shows the SEM backscattered electron images for different levels of cross-contamination. Regions of copper particles, which exhibit greater brightness owing to their increased material density, are marked with circles in the images for reference purposes. In line with the findings from [17] at 0.5 wt.% contamination levels, these particles/precipitates appear as single isolated defects that are likely to be dispersed randomly within the matrix of the material. The enhanced backscattered signal indicates a higher average atomic number and density than the surrounding Al–Si matrix, consistent with Cu-rich metallic or intermetallic inclusions originating from the CuCr1Zr powder and partially dissolving during LPBF. Based on the composition of the powder feedstock, as well as observations reported for similar systems, these particles can be reasonably interpreted as Cu-enriched Al-based phases containing Cu together with minor Cr and Zr, possibly in the form of complex Al–Cu–(Mg)–(Cr/Zr) intermetallics that segregate along cell and eutectic boundaries [45]. Such inclusions introduce a pronounced elastic and plastic mismatch relative to the ductile AlSi10Mg matrix and are therefore expected to act as potent stress concentrators and preferential crack-initiation sites, contributing to the progressive embrittlement observed at higher cross-contamination levels. At higher contamination levels, e.g., 3.0 wt.% contamination, these sites become more numerous and begin to interconnect, while bright copper-rich regions remain distinctly discernible. At the higher investigated (5.0 wt.%) contamination level, only isolated brighter regions remain discernible. The overall microstructure displays a subtle bright sheen, signifying extensive regions enriched with copper that are less distinctly delineated compared to specimens exhibiting lower contamination levels.

## 4. Quality Assessment

**Figure 11:**
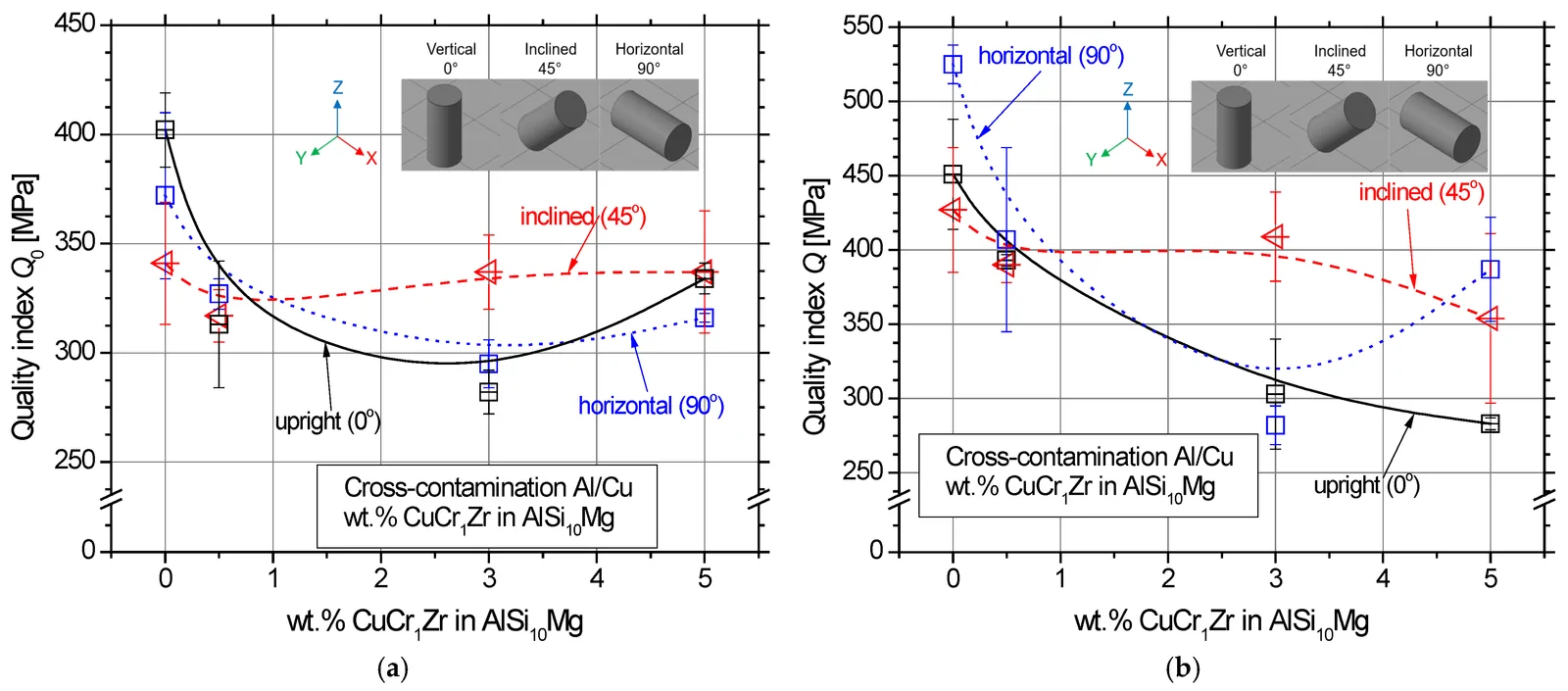
Quality indices (**a**) *Q*_0_ and (**b**) *Q* as a function of the different percentages of CuCr1Zr cross-contamination for specimens printed in different build orientations, namely, upright (0°), inclined (45°), and horizontal (90°).

In general, the presence of contamination increases the material strength while simultaneously reducing its ductility. In other words, the material becomes capable of withstanding higher applied stress, but its ability to undergo plastic deformation decreases. Quality indices are used to balance the combined effects of strength and plastic deformation, providing a more comprehensive evaluation of the mechanical performance of the material. By comparing Figure 11a and Figure 5d, *Q*_0_ appears to follow a similar trend to *W*, indicating that this quality index is strongly influenced by the work-hardening parameter. In contrast, when comparing Figure 11b to Figure 5b, *Q* is mainly dominated by *R*_m_, suggesting that ultimate tensile strength makes the most significant contribution to the behaviour of this quality index.

**Figure 12:**
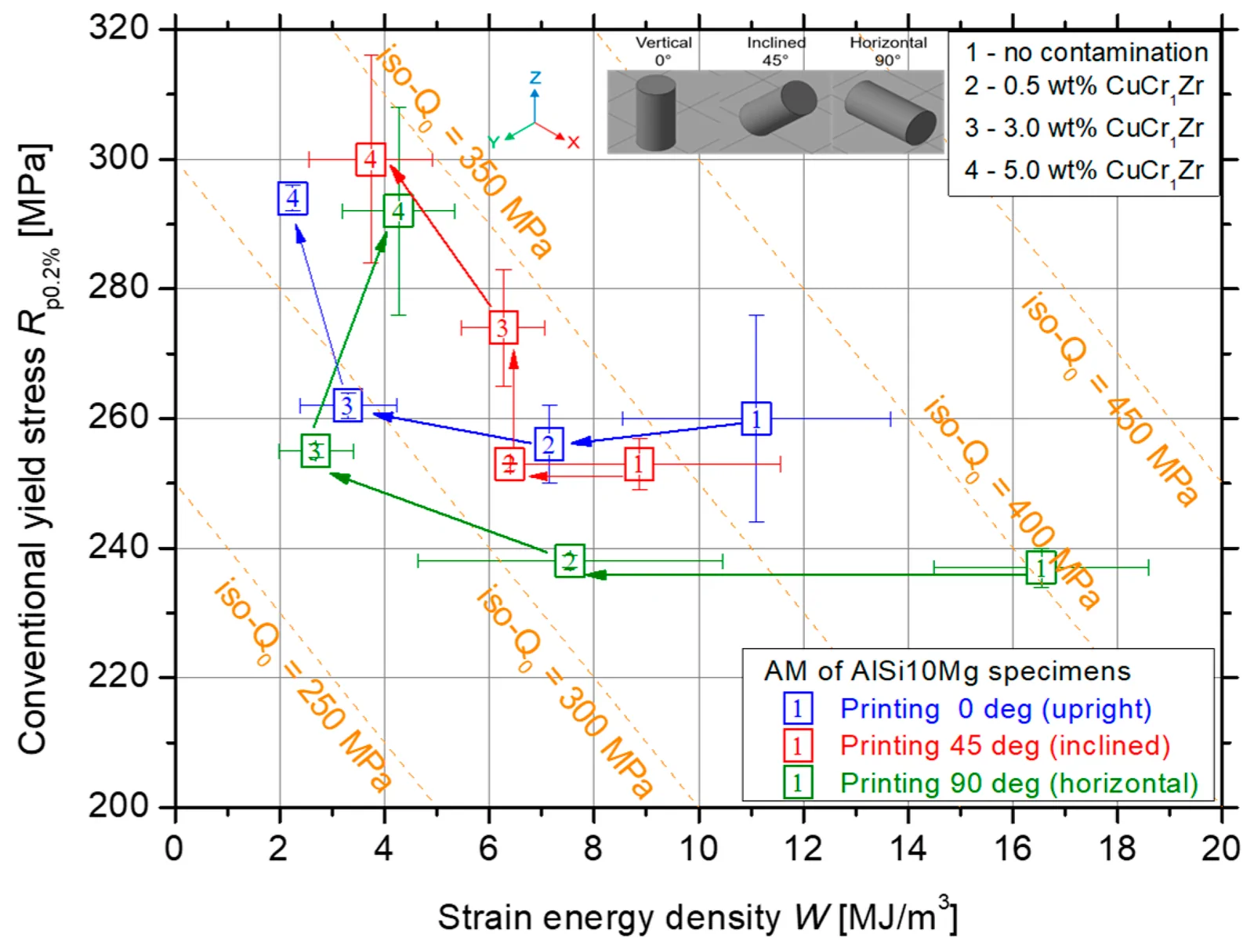
Effect of CuCr1Zr cross-contamination and printing direction on the quality index of additively manufactured AlSi10Mg specimens.

The quality index results for the different cross-contamination levels and printing orientations are presented in Figure 12 as a plot of yield stress (*y*-axis) versus strain energy density (*x*-axis). The horizontal (90°) built orientation with no contamination exhibits the highest quality index (approximately 370 MPa). With increasing contamination level, the quality index for horizontally (90°) printed samples decreases and then levels off at approximately 300 MPa for the highest cross-contamination conditions investigated. The most pronounced reduction in quality is observed for vertically (0°) printed specimens as contamination gradually increases, although a partial recovery appears at the highest contamination level. Finally, the inclined specimens printed at 45° to the build direction maintain an almost constant quality across all contamination levels, with the only noticeable deviation occurring at the low contamination level of 0.5 wt.%. The highest contamination level of 5.0 wt.% leads to a marked reduction in quality for both the upright and horizontal build orientations. In contrast, the inclined specimens remain almost unaffected by cross-contamination, as their quality stays nearly constant across all contamination levels, effectively balancing the loss in tensile ductility against the gain in mechanical strength arising from intermetallic phase formation.

## 5. Conclusions

An experimental investigation, complemented by qualitative analysis, was performed to assess the effect of CuCr1Zr cross-contamination in additively manufactured AlSi10Mg specimens on their microstructural features, tensile mechanical performance, strain-hardening behaviour and quality assessment. The major findings are summarised below:

- CuCr1Zr contamination is responsible for the formation of copper-rich regions in the aluminium matrix that are heavily dependent on the cross-contamination level. Higher contamination levels resulted in a substantial increase in yield stress, accompanied by an essential decrease in tensile ductility.
- The building direction of additively manufactured specimens plays a critical role in the tensile mechanical properties, with horizontally printed samples (90° to build direction) exhibiting superior tensile properties across nearly all contamination levels.
- Two different stages of strain-hardening were evaluated for all investigated tensile specimens, namely, Stage I (easy glide) and Stage II (athermal work-hardening), while Stage III (dynamic recovery) was not noticed.
- Stage I exhibited strain-hardening exponents (~0.18) that were increased with the contamination level. Higher strain-hardening exponents (>0.27) were noticed for Stage II, which decreased with increased cross-contamination level.
- The highest quality index for the non-contaminated specimens was noted for the upright (0°) building direction.
- Quality index decreased for all build directions with increasing cross-contamination level. Despite the lower quality index of the non-contaminated specimens, the inclined (45°) printed specimens presented quality indices that were almost unaffected by the cross-contamination level.

## Data Availability

The original contributions presented in this study are included in the article. Further inquiries can be directed to the corresponding author.
